# Disruption of *Rest* Leads to the Early Onset of Cataracts with the Aberrant Terminal Differentiation of Lens Fiber Cells

**DOI:** 10.1371/journal.pone.0163042

**Published:** 2016-09-15

**Authors:** Hitomi Aoki, Hajime Ogino, Hiroyuki Tomita, Akira Hara, Takahiro Kunisada

**Affiliations:** 1 Department of Tissue and Organ Development, Gifu University Graduate School of Medicine, Gifu, Japan; 2 Department of Animal Bioscience, Nagahama Institute of Bio-Science and Technology, Nagahama, Japan; 3 Department of Tumor Pathology, Gifu University Graduate School of Medicine, Gifu, Japan; Rush University Medical Center, UNITED STATES

## Abstract

REST (RE1-silencing transcription factor, also called Nrsf) is involved in the maintenance of the undifferentiated state of neuronal stem/progenitor cells in vitro by preventing precocious expression of neuronal genes. REST expression was then decreased in developing neurons to down-regulate neuronal genes which allow their maturation. However, the function of REST during neurogenesis in vivo remains to be elucidated because of the early embryonic lethal phenotype of conventional *Rest* knockout mice. In order to investigate the role of REST in ocular tissues, we generated and examined the mice evoking genetic ablation to *Rest* specifically to neural tissues including ocular tissue. We used a *Sox1-Cre* allele to excise the floxed *Rest* gene in the early neural tissues including the lens and retinal primordia. The resulting *Rest* conditional knockout (CKO) and co cntrol mice were used in comparative morphological, histological, and gene expression analyses. *Rest* CKO mice had an abnormal lens morphology after birth. The proliferation of lens epithelial cells was likely to be slightly reduced, and vacuoles formed without a visible increase in apoptotic cells. Although the aberrant expression of late onset cataract marker proteins was not detected, the expression of Notch signaling-related genes including a previously identified REST-target gene was up-regulated around birth, and this was followed by the down-regulated expression of lens fiber regulators such as *c-Maf* and *Prox1*. *Rest* CKO induces a unique cataract phenotype just after birth. Augmented Notch signaling and the down-regulated expression of lens fiber regulator genes may be responsible for this phenotype. Our results highlight the significance of REST function in lens fiber formation, which is necessary for maintaining an intact lens structure.

## Introduction

The transcriptional repressor RE1-silencing transcription factor, REST (also known as neuron-restrictive silencer factor NRSF), was initially discovered as a negative regulator of neuron-specific genes in non-neuronal cells [1,2]. REST is expressed throughout early development and represses the expression of neuronal genes by transcriptionally silencing their promoters in conjunction with CoREST [3]. The target genes of REST include a number of genes encoding ion channels, neurotrophins, synaptic vesicle proteins, and neurotransmitter receptors [4–6].

Previous studies have shown that the expression of *Rest* is down-regulated as neural stem cells (NSCs) differentiate into mature neurons, and is completely silenced in mature adult neurons [7]. The repressor function of REST indicates that it plays a central role in inhibiting the precocious expression of neuronal genes in NSCs. Furthermore, its down-regulation upon the receipt of neuronal differentiation cues has been shown to permit the robust expression of neuronal genes for terminal differentiation [7].

A previous study reported that the targeted mutation of *Rest* in mice resulted in the de-repression of a neuron-specific tubulin gene in a subset of non-neuronal tissues [8]. Although dispensable for embryonic neurogenesis in vivo, *Rest* was indicated to play a role in suppressing the expression of neuronal genes in cultured neuronal precursor cells and developing non-neural tissues [9,10]. *Rest* null mice survive to E9 without obvious morphological defects, by which time all three germs layers and the neural tube forms, clearly demonstrating that neuronal progenitors have the ability to develop *in vivo* in the absence of *Rest*. However, *Rest* null mice die by E11.5, prior to which the growth retardation caused by widespread apoptotic cell death starts at approximately E9.5 [8]. This early embryonic lethality has precluded further analyses of the potential role of *Rest* in the maintenance and differentiation of neural and non-neural cells *in vivo*.

In addition to its involvement in neurogenesis, recent studies have suggested that REST modulates glial lineage elaboration by coupling neurogenesis and gliogenesis [11,12], and the breakdown of these processes accompanies neurodegenerative disorders. Neural crest derived enteric nerve cell specific reduction of acetylcolinesterase was detected in the *Rest* CKO mice to induce the failure of the gut function by underdeveloped cholinergic transmission in the enteric nervous system [13]. The disruption of REST and its target gene interactions has been detected in patients with Alzheimer’s disease [14], microcephaly [15], epileptic seizures [16], Huntington’s Disease [17], Down’s syndrome [18,19]. In these neurodegenerative disorders, REST dysfunction has been suggested to cause the aberrant expression of various genes including neuronal genes.

Eye development is attained by close interactions between retinal and lens primordia [20]. Since the neural retina is part of the central nervous system and the lens is one of the sensory placode-derived tissues, their development may be under the control of the *Rest* gene. Thus, using our established *Rest* conditional knockout system in mice, we attempted to identify a possible ocular phenotype caused by the lack of REST expression. This approach revealed a novel function of *Rest* in lens development.

## Materials and Methods

### Animals

Mice were handled in compliance with the ARVO Statement for the Use of Animals in Ophthalmic and Vision Research. All animal experiments were approved by the Animal Research Committee of the Gifu University Graduate School of Medicine. *Rest*^2lox/2lox^ mice were generated from the *Rest*^2lox/+^ ES cell line as described previously [9,21]. *Sox1-Cre* mice [9,22] were bred with *Rest*^2lox/2lox^ mice, *Rosa26R-EYFP* mice [23], or *Rosa26R-LacZ* mice [24] to generate compound transgenic mice. The *Rest*^2lox/+^ ES cell line was generated from F1 (129SvJae x C57BL/6; V6.5 line) mouse ES cells. *Sox1-Cre* mice, *Rosa26R-EYFP* mice, and *Rosa26R-LacZ* mice had the C57BL/6 background. The results obtained for *Rest* CKO mice (*Rest*^2lox/2lox^; *Sox1-Cre+*) were compared with those for *Rest*^2lox/2lox^; *Sox1-Cre-* mice and *Rest*^+/+^; *Sox1-Cre+* mice as a control. *Sox1-Cre*+; *Rosa26R-EYFP* embryos were dissected at E9.5, EYFP signals were examined under a fluorescent stereomicroscope (SZX16, Olympus), and digital images were captured with an Olympus DP70.

### LacZ staining

*Sox1-Cre*+; *Rosa26R-LacZ* embryos were dissected at E10.5. The methods used for LacZ staining have been described in detail previously [25,26]. Briefly, embryos were fixed for 10 min in 2% paraformaldehyde supplemented with 0.2% glutaraldehyde and 0.02% Tween-20. After three washes in PBS, embryos were stained overnight at 37°C in 10 mM phosphate buffer (pH 7.2) containing 1.0 mM MgCl_2_, 3.1 mM K_4_ [Fe(CN)_6_], 3.1 mM K_3_[Fe(CN) _6_], and 2 mg/mL X-Gal. The staining reaction was stopped by washing in PBS. Specimens were postfixed overnight with phosphate buffer containing 4% formaldehyde (pH 7.2). *Sox1-Cre*+; *Rosa26R-LacZ* eyes from P0 pups were dissected and embedded in OCT compound and used for frozen sections. Frozen sections were stained using the same protocol as that for embryos.

### Histology and immunohistochemistry

The eyes were enucleated and fixed by immersion overnight in phosphate buffer containing 4% formaldehyde (pH 7.2). The methods used for histological analyses have been described previously [9]. Briefly, specimens were dehydrated with ethanol, soaked in xylene, and embedded in paraffin. Serial sections were prepared at a thickness of 3 μm using a Leica RM2125RT microtome (Leica Microsystems Inc., Bannockburn, IL) and stained with hematoxylin and eosin (HE). We used at least four eyes from two mice for each of the indicated genotypes and time points shown in Table 1. At least five and three sections for HE staining and immunohistochemistry, respectively, were prepared from one eye at appropriate intervals.

**Table 1:** The penetrant of the lens phenotype in *Rest* CKO mice.

genotype	*Rest* CKO			control		
stage	abnormal (No)	normal (No)	incidence (%)	abnormal(No)	normal (No)	incidence (%)
No. of pups at E13.5	0	7	0	0	11	0
No. of pups at E14.5	0	8	0	0	11	0
No. of pups at E15.5	0	9	0	0	14	0
No. of pups at E16.5	0	8	0	0	11	0
No. of pups at E17.5	0	7	0	0	5	0
No. of pups at P0	17	6	73.91	0	39	0
No. of pups at P1	12	3	80	0	8	0
No. of pups at P3	11	1	91.67	0	13	0
No. of pups at P4	9	0	100	0	6	0
No. of pups at P5	6	0	100	0	7	0
No. of pups at P7	3	0	100	0	3	0
No. of pups at P10	4	0	100	0	2	0
No. of pups at 2W	3	0	100	0	3	0
No. of pups at 4W	4	0	100	0	2	0
No. of pups at 8W	21	0	100	0	23	0

A HISTOFINE Kit (Nichirei Bio Science, Japan) or VECTASTAIN ABC System (Vector laboratories, CA, USA) was used for immunohistochemistry according to the manufacturer’s protocols with 3,3’-diaminobenzidine (DAB). Specimens were examined under an Olympus BX-51 microscope (Olympus, Japan). Images were captured with an Olympus DP70 digital camera.

The primary antibodies used in this study were as follows: anti-mouse Ki67 (1:500; TEK3, Dako-Cytomation, Glostrup, Denmark), anti-Prox1 (1:500; Millipore, MA, USA), anti-gamma Crystallin (1:1000; Bioss, MA, USA), anti-c-Maf (1:500; Novus, CO, USA), and anti-smooth muscle actin (1:500; SMA, Dako).

Histological evaluations were performed with the support of two experienced pathologists (H.T. and A.H.) who were blinded to the experimental data.

### In situ terminal dUTP-biotin nick end labeling of DNA fragments (TUNEL) method

TUNEL staining was performed to detect apoptotic cells as described previously [25,26]. After being incubated in 20 **μ**g/ml proteinase K (Sigma), the serial sections used for HE staining were immersed in TDT buffer (30 mM Trizma base, pH 7.2, 140 mM sodium cacodylate, and 1 mM cobalt chloride). TDT and biotinylated dUTP (both from Roche) were diluted with TDT buffer at concentrations of 0.15 e.u./ml and 0.8 nmol/ml, respectively. The solution was placed on the sections and then incubated at 37°C for 60 min. The sections were covered with streptavidin peroxidase (DAKO, Carpinteria, CA, USA) and stained with DAB as a substrate for peroxidase. Counterstaining was performed using Mayer’s hematoxylin.

### Gene expression analysis

Whole lenses were dissected from enucleated eyes of each embryo or mouse at the indicated embryonic or postnatal days and used for total RNA purification. 6, 15, 5, 4, 3, and 6 mice of each respective genotype were used to prepare RNAs at E13.5, E15.5, E17.5, P1, P7 and 10W, respectively. Total RNA was prepared using the RNeasy Plus Mini Kit (Qiagen) according to the manufacturer’s instructions. First-strand cDNA was synthesized from 1 μg of total RNA using the SuperScript First-Strand Synthesis System (TAKARA BIO, Japan) with oligo dT primers. Real time PCR was performed using gene-specific primers and SYBR Premix Ex Taq (TAKARA BIO) with Thermal Cycler Dice Real Time System (TAKARA BIO). Real-time quantitative PCR was performed using the relative standard curve method to quantify the target gene expression. For normalization,*β-actin* was used. The primer sequences used in qRT-PCR analyses were obtained from the PrimerBank (http://pga.mgh.harvard.edu/primerbank/).

### Statistical analysis

Statistical differences were assessed using the Student’s *t*-test. P values of <0.05 or <0.01 were considered significant. Error bars in graphs denote s.d.

## Results

### Conditional ablation of *Rest* by *Sox-Cre* results in a postnatal cataract-like phenotype

**Fig 1:**
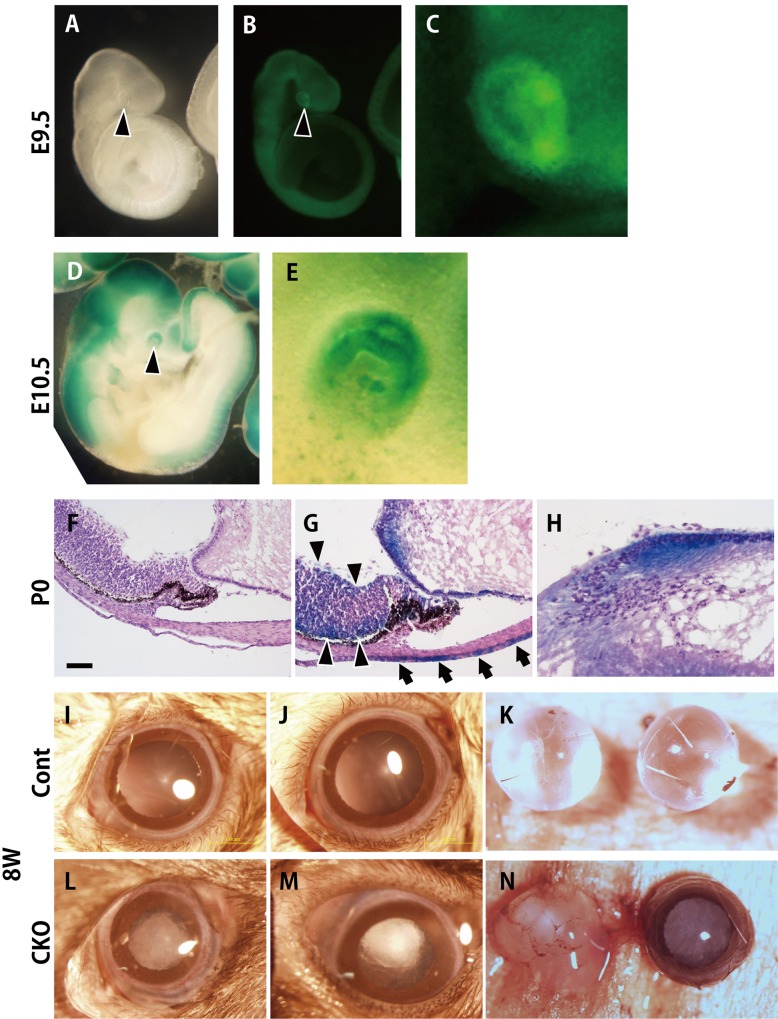
*In vivo* genetic ablation of *Rest* in developing embryos leads to cataract-like symptoms. (A-H) The direct detection of YFP or LacZ staining in *Sox1*-*Cre*-positive cells. The direct detection of YFP in *Sox1*-*Cre*-positive cells in *Sox1*-*Cre+*; *Rosa26R-EYFP* mice at E9.5 (A-C). LacZ staining of *Sox1*-*Cre*-positive cells in *Sox1*-*Cre+*; *Rosa26R-LacZ* mice at E10.5 (D and E). Arrowheads indicate optic vesicles. (F-H) LacZ staining of *Sox1*-*Cre* positive cells in *Sox1*-*Cre+*; *Rosa26R-LacZ* mice at P0. LacZ-positive cells were observed in the lens epithelium (H) as well as the corneal epithelium (arrow) and neural retina (arrowhead) of *Sox1*-*Cre+*; *Rosa26R-LacZ* mice at P0 (G). *Sox1*-*Cre-*; *Rosa26R-LacZ* control section was shown in F. (I-N) Exterior view of the eye and lens. (I, J, L, M) Exterior view of the eye. Lens opacity was observed in 8-week-old *Rest* CKO mice (L, M), but not in control mice (I, J). Lenses dissected from the eyeballs of control mice maintained their shape and clarity (K). An eyeball (right in N) dissected from a *Rest* CKO mouse and a lens after being dissected from another eyeball (Left in N) were also shown (N). Lenses dissected from *Rest* CKO eyes readily collapsed after their removal from the eyes (left in N).

A previous study revealed that mice lacking the *Rest* gene died during early embryonic development [8]. In order to examine the effects of a *Rest* deletion in the central nervous system *in vivo*, we established mice containing floxed *Rest* alleles and *Sox1-Cre* alleles (*Rest*^2lox/2lox^; *Sox1-Cre+*, named *Rest* CKO) [9,21]. *Sox1* is a transcription factor gene whose expression occurs around E7.5 to E8.5 in the neural tube [22] and subsequently persists in the developing neural retina and retinal pigment epithelium [27]. *Sox1* expression also starts in the invaginating lens placode by E10.5 [23,28]. Consistent with these findings, the expression of *Sox1-Cre*-induced YFP started at approximately E9.5 in ocular tissue (Fig 1A–1C) and the *Sox1-Cre*-induced expression of LacZ was detected at E10.5 in the lens placode (Fig 1D and 1E). Histological analyses also showed that the majority of lens epithelial cells as well as neural retinal and corneal epithelial cells were LacZ-positive at P0, indicating the expression of Cre recombinase driven by the *Sox1* promoter in the lens epithelium (Fig 1F–1H).

We found that *Rest* CKO mice were born without an apparent abnormal morphology as previously reported [9]. However, 8 weeks after birth, mice developed severe lens opacity, which blocks or changes the entry of light, affecting vision and a **cataract** is characterized by **opacities** (Fig 1I, 1J, 1L and 1M). Furthermore, these lenses were so fragile that they easily broke down after being removed from the eye (Fig 1K–1N).

### Vacuole formation and reduced proliferation of lens epithelial cells in the *Rest* CKO lens

**Fig 2:**
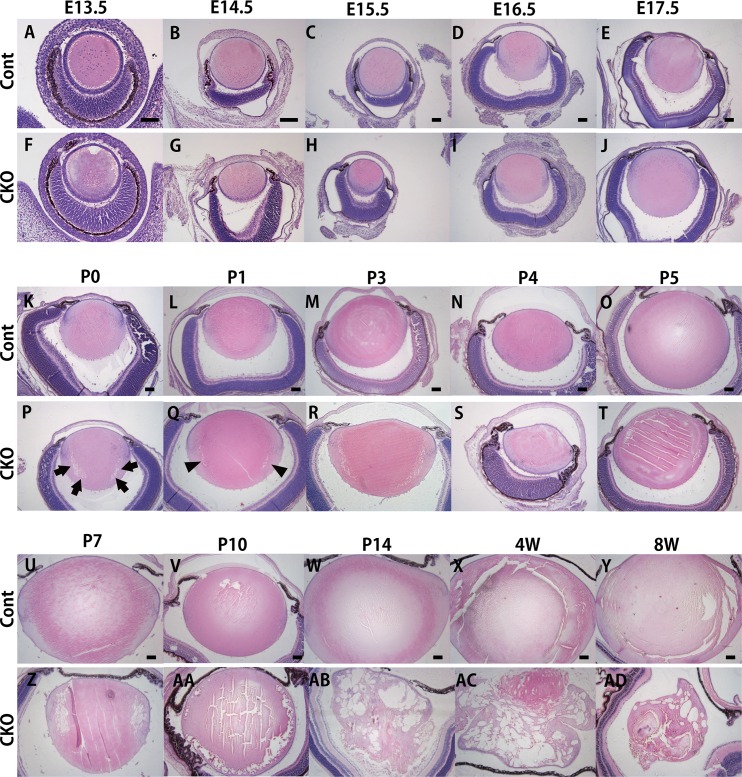
The sequential histology of the lens at the indicated time points in *Rest* CKO mice and their control littermates. Lenses at E13.5 (A, F), E14.5 (B, G), E15.5 (C, H), E16.5 (D, I), E17.5 (E, J), P0 (K, P), P1 (L, Q), P3 (M, R), P4 (N, S), P5 (O, T), P7 (U, Z), P10 (V, AA), P14 (W, AB), 4W (X, AC), and 8W (Y, AD) in *Rest* CKO mice (F-J, P-T, and Z-AD) and their control littermates (A-E, K-O, and U-Y) were shown. Lenses from *Rest* CKO adult mice clearly showed an abnormal morphology in the lens cortex and/or bow region, where newly formed secondary lens fibers are present, by HE staining. Arrows and arrowheads indicate the formation of vacuoles and gaps in lens fibers, respectively. Scale bars represent 50 μm.

**Fig 3:**
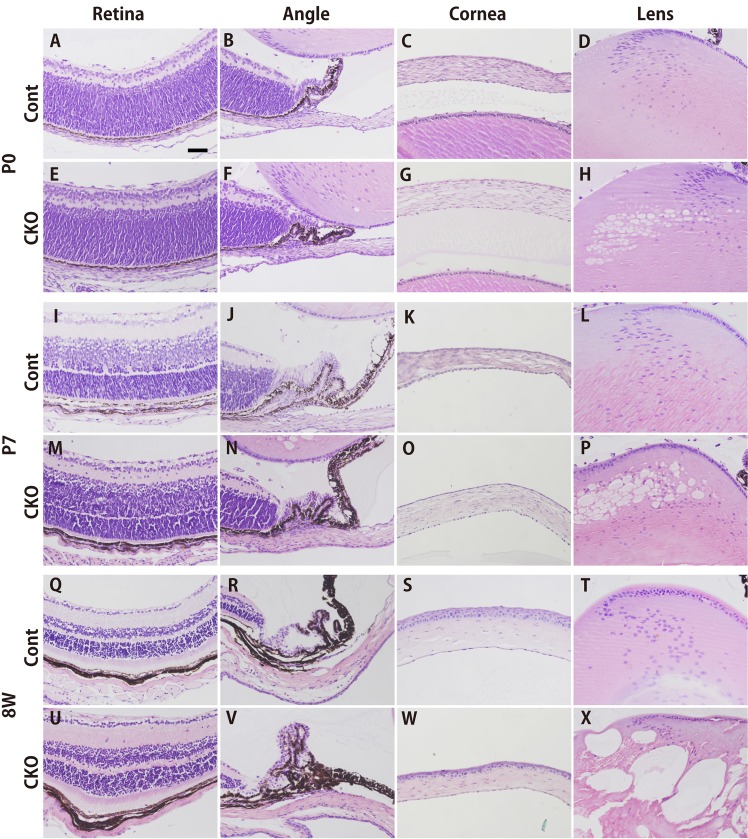
*In vivo* genetic ablation of *Rest* leads to abnormal lens differentiation. Ocular tissues at P0 (A-H), P7 (I-P), and 8W (Q-X) in *Rest* CKO mice (E-H, M-P, U-X) and their control littermates (A-D, I-L, Q-T). The retina (A, E, I, M, Q, U), angle (B, F, J, N, R, V), cornea (C, G, K, O, S, W), and lens (D, H, L, P, T, X) were shown. The scale bar represents 50 μm.

In order to characterize the *Rest* CKO phenotype at the histological level, we performed a HE staining analysis of eye tissues dissected at embryonic days (E) 13.5, E14.5, E15.5, E16.5, and E17.5, and postnatal days (P) 0, P1, P3, P4. P5, P7, P10, P14, 4 weeks (W), and 8W (Fig 2). The eyes of *Rest* CKO mice started to exhibit an abnormal lens morphology after birth (lens from a *Rest* CKO mouse in Fig 2P–2T and 2Z–2AD and that of a control in Fig 2K–2O and 2U–2Y), but not during embryogenesis (lens from a *Rest* CKO mouse in Fig 2F–2J and that of a control in Fig 2A–2E). The number and incidence of the lens phenotype were summarized in Table 1. Dysplasia appeared as the formation of a vacuole (arrows in Fig 2P) or gaps (arrowheads in Fig 2Q) in the peripheral part of the lens fiber. The size and number of these vacuoles or gaps then increased, while the central part of the lens fiber mass began to show an irregular alignment, which was revealed as deformed layer structures (lens from a *Rest* CKO mouse in Figs 2P and 3H and that of a control in Figs 2K and 3D).

On the other hand, other ocular tissues, the retina, cornea, and uvea, including the choroid, iris, and ciliary body, did not show any morphological abnormalities in their major structural components (eye from a *Rest* CKO mouse in Fig 3E–3G and that of a control in Fig 3A–3C). The lenses of all *Rest* CKO mice started to show amorphous and disorganized lens fibers by P7 (lens from a *Rest* CKO mouse in Fig 3P and that of a control in Fig 3L) and large vacuoles 8 weeks after birth (lens from a *Rest* CKO mouse in Fig 3X and that of a control in Fig 3T), without any obvious histological abnormalities in other ocular tissues (eye from a *Rest* CKO mouse in Fig 3M–3O and 3U–3W and that of a control in Fig 3I–3K and 3Q–3S).

### *Rest* CKO is likely to interferes with the proliferation of lens epithelial cells

**Fig 4:**
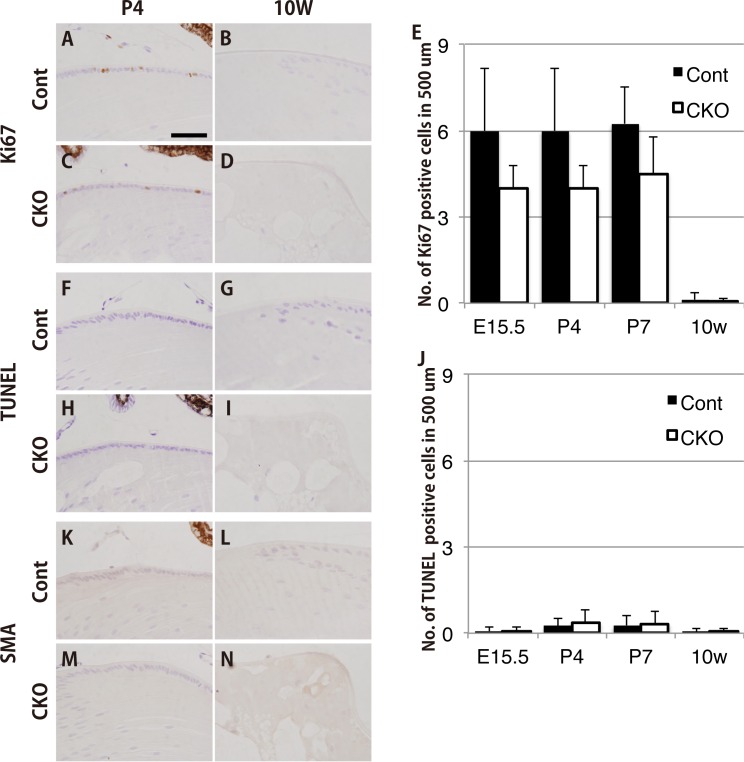
Cell proliferation, apoptosis, and cataract marker protein expression in *Rest*–deficient lenses *in vivo*. (A-E) An immunohistochemical analysis of Ki67-positive proliferating cells. No significant differences were observed in the distribution of Ki67-positive cells in the surface layer of the lens capsule, regardless of their genotypes (A-D). Although not statistically significant, the number of Ki67-positive cells/length in the surface layer of the lens capsule of *Rest* CKO mice was slightly lower than that of their control littermates (E). (F-J) A histological analysis of TUNEL-positive apoptotic cells. Almost no TUNEL-positive cells were detected in the surface layer of the lens capsule, regardless of their genotypes (F-I). No significant difference was observed in the number of TUNEL-positive cells between *Rest* CKO mice and their control littermates (J). (K-N) Immunohistochemical staining for the proteins known to be associated with age-related cataracts in humans. Virtually no SMA-positive cells were detected in the surface layer of the lens capsule, regardless of their genotypes. The scale bar represents 50 μm.

In order to investigate the mechanisms underlying vacuole or gap formation, the proliferation of lens epithelial cells was examined in the E15.5, P4, P7, and 10W lenses of CKO mice. A slight decrease in the number of proliferating Ki67-positive cells was observed in the lens epithelial layer at each time point (Fig 4A–4E), however, it’s not significant. This suggest that it might be hard to explain only in such small decrease in lens epithelial number would result in the rather major disruption of lens structure. Then, We also performed a TUNEL assay to detect apoptotic changes in the E15.5, P4, P7, and 10W lenses of CKO mice. However, no detectable increase in the number of apoptotic cells was observed in the lens epithelial layer, indicating that apoptosis did not play a major role in the deformed phenotype (Fig 4F–4J).

### Expression of lens protein markers in *Rest* CKO mice

**Fig 5:**
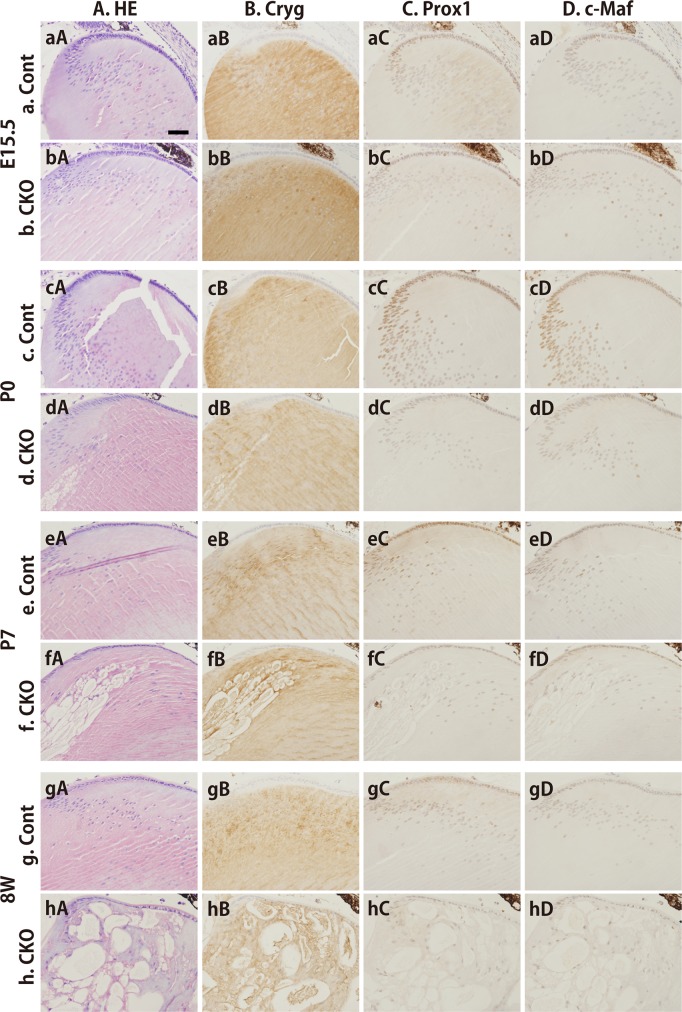
Immunohistochemical detection of lens marker proteins in lenses from a *Rest*–deficient mouse *in vivo*. HE staining (A) and immunohistochemical staining with antibodies against Cryg (B), Prox1 (C), and c-Maf (D) at E15.5 (a and b), P0 (c and d), P7 (e and f), and 8W (g and h) in lenses from *Rest* CKO mice (b, d, f, h) and control littermates (a, c, e, g). HE staining clearly showed disorganization after birth (A). Cryg was strongly expressed in both genotypes at all time points (B). The expression levels of Prox1 and c-Maf in lenses were lower in *Rest* CKO mice than in the controls after birth (C and D). The scale bar represents 100 μm.

We examined the expression patterns of commonly used lens protein markers such as Cryg, Prox1, and c-Maf by immunostaining at various ages (Fig 5 and S1 Fig). The expression of Cryg was constantly abundant in lenses from *Rest* CKO and control mice from E15.5 to 8 weeks after birth (Fig 5B and S1B Fig), while that of Prox1 and c-Maf was slightly lower in *Rest* CKO mice than in control mice from P0 and P7 (Fig 5C and 5D and S1C and S1D Fig).

Previous studies have shown that the increased expression of SMA was only seen in nuclear cataract but not in cortical cataract, the major form of age-related opacities [29,30]. Thus, we examined expression profiles of SMA in *Rest* CKO and control lenses. However, we did not detect any signal in either tissue (Fig 4K–4N), indicating that SMA is not associated with the cataract-like phenotype of *Rest* CKO mice.

### Expression profiles of REST target genes in the developing lens

**Fig 6:**
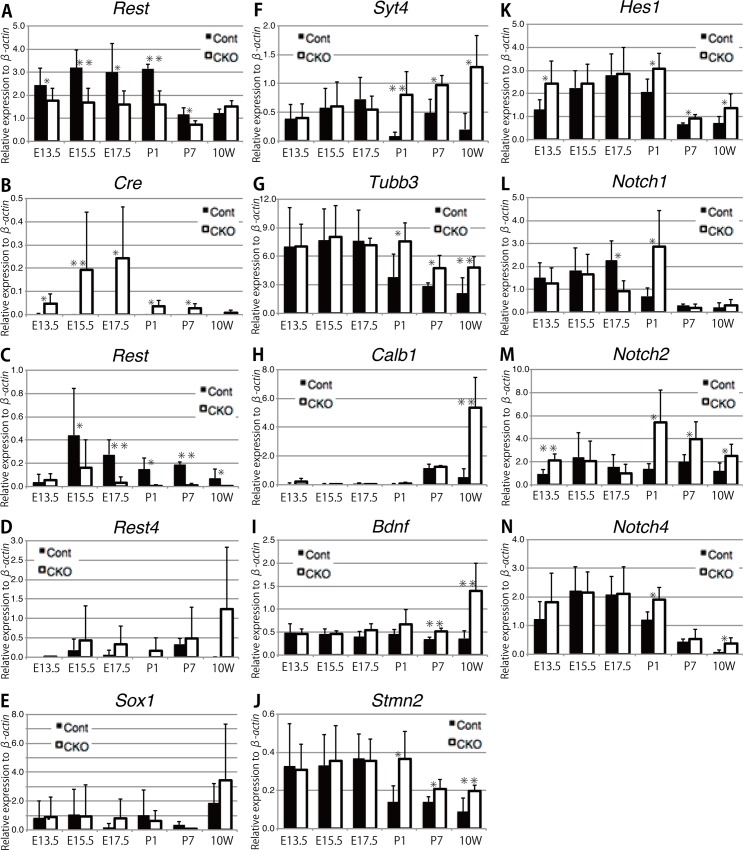
A gene expression analysis of *Rest*, *Cre*, and *Rest* target genes, and Notch signaling-related genes in the *Rest*-deficient lens. The mRNA levels of *Rest* (A), *Cre* (B), *Rest* and *Rest4* (C and D, detected by the previously reported primers^29^), *Sox1* (E), *Rest* target genes (F-J), and the indicated Notch signaling-related genes (K-N) in *Rest* CKO and control lenses were measured by quantitative RT-PCR. Transcript levels were normalized to *β-actin*. Data are presented as average values with s.d. of more than three to fifteen independent samples. *; p<0.05, **; p<0.01.

In order to elucidate the molecular mechanisms responsible for abnormal lens development in *Rest* CKO mice, we first assessed the expression of *Rest* mRNA from dissected developing lenses. The primer pair was set inside of the floxed exon 4 of *Rest* gene. As shown in Fig 6A, *Rest* mRNA levels were significantly reduced in E13.5 to E17.5 *Rest* CKO lenses and P1, P7 *Rest* CKO lenses. *Rest* mRNA detected in CKO mice might be originated from the contaminated non-lens tissues. When the other primer pairs [31] was used, still *Rest* mRNA was detected from the control, *Rest* mRNA levels were significantly reduced in E15.5 to 10 week *Rest* CKO (Fig 6C). *Cre* mRNA was detected as early as E13.5 and a concomitant reduction in *Cre* mRNA expression levels of at least 10-fold was observed (Fig 6B). No significant difference was observed in the expression of *Sox1* between control and *Rest* CKO lenses (Fig 6E). Therefore, the phenotypic change observed in *Rest* CKO mice (Fig 1I–1N) appeared far later than the start of reductions in *Rest*. The expression of *β-actin*, which was determined as a quantitative control, remained constant in each developmental stage (S2A Fig). As expected, the expression of some neuronal genes known as targets of REST, such as *Syt4*, *Tubb3*, *Calb1*, *Bdnf*, and *Stmn2* was significantly up-regulated after birth in the *Rest* CKO lens (Fig 6F–6J). The expression of *REST* has previously been investigated in the lens [31,32]. They demonstrated that the expression of *REST* and a splice isoform of REST/NRSE called REST4 lacking a transcription repression domain predominated during lens fiber differentiation. After investigating *Rest4* expression using the primer pair specifically detect *Rest4* transcript [31], we found that the expression of *Rest4* was significantly up-regulated in *Rest* CKO lens from E15.5 to 10 week (Fig 6D). This suggests that the ablation of the floxed *Rest* DNA induced by *Sox1-Cre* affect the alternative splicing pattern of *Rest* mRNA to preferentially generate *Rest4* transcript.

Genome-wide mapping of *in vivo* protein-DNA interactions previously revealed that *Hes1* is an immediate target of the REST protein [33]. Genome-wide identification of novel REST binding sites by ChIP-PET technology also revealed that *Hes1* has a REST binding site and is one of the potential target genes (Dataset S4 in Johnson et al., 2008)[34]. As shown in Fig 6K, the expression of *Hes1* was greater at birth in *Rest* CKO than in the control, which was consistent with the transcription repressor function of REST for the *Hes1* gene [33].

*Hes1* is an important key factor involved in Notch signaling. Therefore, we examined the expression of the genes constituting the Notch signaling cascade (Fig 6L–6N and S2B–S2D Fig). Among them, the expression of *Notch1* was significantly up-regulated in the P1 lens of mice, while that of *Notch2* and *Notch4* was significantly up-regulated in P1, P7, and adult lenses (Fig 6L–6N). The expression of Notch ligands such as *Jagged2* and *Dll1* was maintained at low levels in the control, whereas that of *Jagged2* was significantly and gradually up-regulated with age in the *Rest* CKO lens after birth and that of *Dll1* was also significantly up-regulated in the *Rest* CKO lens after P1 (S2B–S2D Fig). These results suggest that the enhanced expression of *Hes1* by the *Rest* CKO status in the lens activates Notch signaling through an increase in the Notch receptor and their ligands. The involvement of Notch signaling has been directly tested in the developing lens and Notch signaling has been shown to control the timing of primary fiber cell differentiation and is essential for secondary fiber cell differentiation [35]. Gain- or loss-of-function mutations in Notch signaling lead to the formation of a dysgenic lens, and profound postnatal degeneration has been reported in the lens of an induced Notch loss-of-function mutant [36,37].

Previous findings have also suggested that Cyclin D1 and Cyclin D2 as well as the p27 (Kip1) cyclin-dependent kinase (CDK) inhibitor act downstream of Notch signaling, thereby defining multiple critical functions for this pathway during lens development [35]. Therefore, we assessed the expression of CDK inhibitor genes such as *p21* and *p27*. The expression of both of these genes was maintained at low levels in the control (S2A and S2B Fig), whereas that of *p27* was significantly and gradually up-regulated with age in the *Rest* CKO lens after birth, similar to *Jagged2* (S3B Fig), and that of *p21* was also significantly up-regulated in the *Rest* CKO lens at all time points, except for E17.5 (S3A Fig). On the other hand, the expression of *p57* and *CyclinD1* tended to be gradually down-regulated in both control and *Rest* CKO lens (S3C and S3D Fig). We herein demonstrated that augmented Notch signaling under the influence of the *Rest* CKO status was sufficient to interfere with normal lens fiber development after birth.

### Expression profiles of genes involved in lens fiber differentiation

We examined the expression of genes involved in lens development according to the classification by Ogino et al. (2012)[37]. The expression level of lens-specification gene, *Pax6* was lower before birth and higher after birth in *Rest* CKO lens than in the control lens (S2E Fig). The expression level of *Sox2* was lower at E17.5 and higher at P1 in *Rest* CKO lens than in the control lens (S2F Fig). Another lens lineage-specification gene, *Six3* was also down-regulated in most of the developmental stages of the *Rest* CKO lens (S2G Fig). These fluctuations in each lens-specification gene by *Rest* CKO were very small and the lens-specification process was not likely to be affected by the conditional ablation of *Rest*, at least in the embryo (Fig 2).

**Fig 7:**
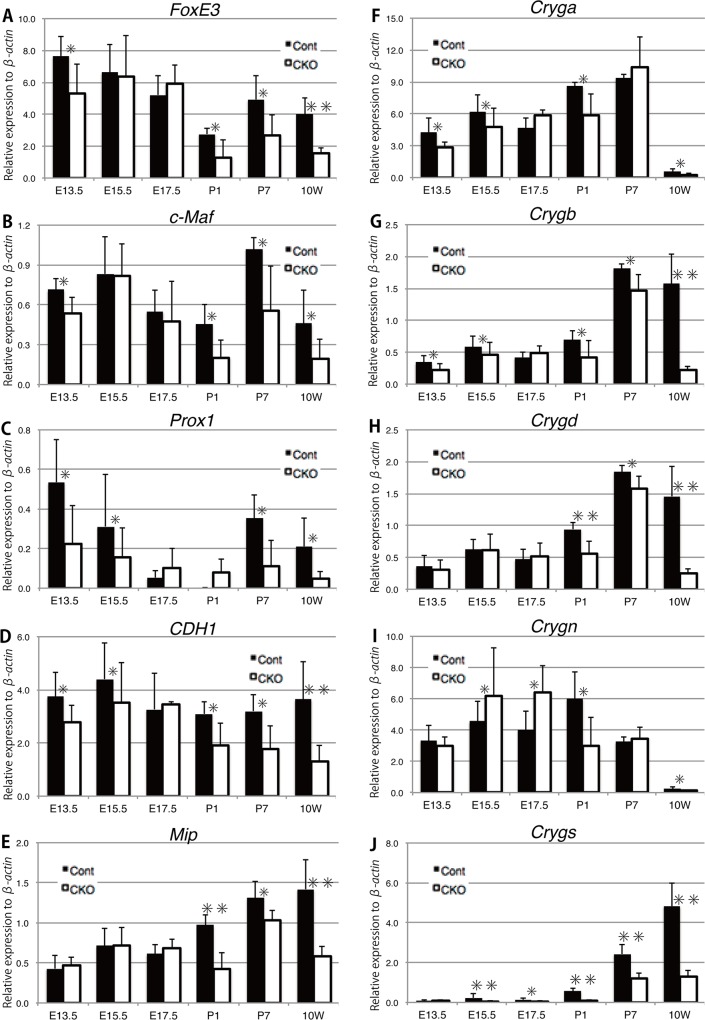
Gene expression of lens markers in various developmental stages of *Rest* CKO mice. The mRNA levels of each gene in lenses dissected from the various developing stages of *Rest* CKO and their control mice were measured by quantitative RT-PCR. Transcript levels were normalized to *β-actin* levels. Data are presented as average values with s.d. of more than three to fifteen independent samples. *; p<0.05, **; p<0.01.

The expression of *FoxE3*, a differentiation gene in the lens epithelium, showed a gradual reduction with age in the control lens, but a significant reduction after birth in the *Rest* CKO lens (Fig 7A). Although the expression of lens fiber-differentiation genes including *c-Maf* and *Prox1* did not exhibit constant changes in the control lens, that of *c-Maf* was significantly down-regulated in the *Rest* CKO lens (Fig 7B) while that of *Prox1* was also down-regulated in the *Rest* CKO lens, except around birth (Fig 7C), suggesting that the cataract phenotype may be caused by changes in lens-differentiation gene expression. Reduction of Prox1 protein in the developing lens after E16.5 [38] may be corresponding to reduction of *Prox1* mRNA observed after E17.5 to P1 (Fig 7C). Sharply FGF dependent expression of Prox1 protein in the lens fiber cells [39] may be related with the observed complex changes of *Prox1* mRNA. The down-regulated expression of *E-cadherin* (*CDH*) and *Mip* was observed after P1 (Fig 7D and 7E). *CDH* was constantly expressed in the control, whereas that in *Rest* CKO lens was significantly down-regulated after birth (Fig 7D). On the other hand, the expression of *Mip* was gradually up-regulated in control with age, but in *Rest* CKO lens, *Mip* expression was significantly reduced in comparison with that of control mice after birth (Fig 7E). The expression of a series of *gamma-crystallin* genes was also slightly down-regulated in *Rest* CKO mice after P1 (Fig 7F–7J). Decreases in these genes required for the function and maintenance of lens fiber cells may have ultimately led to the formation of vacuole-like structures in the *Rest* CKO lens.

We used *Sox1-Cre* to knockout *Rest*, and this is a knockin mutation that destroys one allele of *Sox1* [22] and may change Sox1-dependent gamma crystallin expression [28]. In order to exclude the possibility that Sox1 haploinsufficiency interferes with lens developmental studies or causes the observed reduction in gamma crystallin transcripts shown in Fig 7, we also analyzed mice with *Rest*^+/+^, *Rest*^2lox/+^, and *Rest*^2lox/2lox^ alleles with or without Sox1-Cre allele (S4 Fig). A gene expression analysis using the lenses from Cre-positive controls and *Rest* CKO mice revealed that *Rest* expression levels in the lens were significantly lower in *Rest* CKO mice than in Cre-positive controls, whereas, *Rest4* levels were not as described previously (S4A and S4B Fig). *Cre* expression was constantly high in mice with *Sox1-Cre*, irrespective of the *Rest* CKO allele while that in mice without *Sox1-Cre* was constantly low irrespective of the *Rest* CKO allele (S4C Fig). The expression levels of *Rest* target genes, such as *Tubb3* and *Syt4*, were significantly higher in homozygous *Rest*^2lox/2lox^ CKO mice than in *Rest*^2lox/+^ or *Rest*^+/+^ mice in the presence of *Sox1-Cre* allele. In the absence of *Sox1-Cre* allele, expression of these genes was rather constant in each *Rest* CKO allele (S4D and S4E Fig). The expression levels of *Hes1* in lenses was also significantly higher in homozygous *Rest*^2lox/2lox^ CKO mice than in *Rest*^2lox/+^ or *Rest*^+/+^ mice in the presence of *Sox1-Cre* allele (S4F Fig). The expression level of *Sox2* in lenses was significantly higher in homozygous *Rest*^2lox/2lox^ CKO mice at P0 (S4H Fig). No statistically significant differences of *Sox1* expression was observed in each *Rest* CKO allele (S4G Fig). At P0, *Sox1* expression levels were markedly lower in *Rest*^2lox/2lox^ mice than in *Rest*^+/+^ mice for unknown reasons. We speculated that this may have been caused by the differences in the mouse line because we maintained the *Sox1-Cre* mouse line and *Rest*^2lox/2lox^ mice line with and without the *Sox1-Cre* allele separately. While we cannot completely exclude the possibility that the phenotype we observed was the indirect, non-cell autonomous effect of *Rest* CKO including the changes of gene expression caused by the stress evoked by the vacuole formation, these results support conditional *Rest* ablation decreasing the genes related to lens differentiation and causing lens abnormalities.

## Discussion

The REST repressor complex has been shown to regulate the differentiation of neuronal progenitors to mature neurons, during which the gradual loss of REST repressor complex binding with the target RE1 site ensures the up-regulated expression of target neuronal genes [7]. Although the embryonic lethal nature of *Rest* gene knockout mice has hampered investigations on the roles of REST in the late developmental stage, we generated *Rest* CKO mice and demonstrated that *Rest* played a role in the differentiation and maturation of lens fiber cells.

*Sox1*-directed Cre expression started as early as E7.5 in the neural plate of the embryo and E10.5 in developing lens cells. Furthermore, although the expression of *Rest* was detected in the developing lens by E13.5, a detectable change in *Rest* CKO in the lens that was first observed around birth, suggesting that the indispensable function of REST in lens development was exerted in the later developmental stage. The eyes of all *Rest* CKO mice developed severe lens opacity 8 weeks after birth and the rupture of the lens capsule is easily observed once removed from the eye. This defect in *Rest* CKO mice appeared to be restricted to the lens structure, without any morphological malformation in other ocular tissues; however, further detailed analyses of gene and protein expression profiles and the distribution of each cell type constituting eye tissues are warranted. The first detectable symptom, a small vacuole-like gap in the posterior part of the lens around birth, was not preceded by an apoptotic reaction until the progression of symptoms to the adult.

A quantitative RT-PCR analysis suggested the activation of Notch signaling in the lens of *Rest* CKO mice. A ChIP-PET analysis of REST target genes revealed the presence of REST target cis elements, named the RE1 site, inside or close to genes such as *β-Catenin*, *Hes1*, *Jagged2*, and *p21* [34], and the expression of all these genes was up-regulated in the *Rest* CKO lens (S3G Fig, Fig 6K, S2C Fig, S3A Fig, respectively). Although the precise mechanisms underlying the increase in Notch signaling in the *Rest* CKO lens requires further investigation based on a previous study that identified *Hes1* as an upstream negative regulator of *Rest* [40], REST may affect Notch signal-related genes by directly binding to their RE1 site. The involvement of Notch signaling was directly examined in the developing lens by mutated Notch signaling molecules as early as E11 [41]. However, in the present study, REST was responsible for a deleterious Notch signaling defect just after birth, suggesting the importance of Notch signaling even for the later stage of lens morphogenesis. It is important to note that the *Sox1-Cre* system also induces the deletion of *Rest* in developing retinal tissues and this may indirectly influence the development of closely associated lens tissues. However, any possible defects caused by *Rest* CKO in the retina, as is expected by the influence of Notch signaling in the maintenance of an retinal progenitor cell population and the proper development of retinal cells including photoreceptor and ganglion cells [42], was never observed in our *Rest* CKO mice, indicating the lens cell-autonomous nature of the *Rest* CKO phenotype.

Under the condition that Notch signaling is augmented by the targeted deletion of *Rest*, reductions in *FoxE3* gene expression regulating lens cell differentiation were observed in contrast to the reported increase after the activation of Notch signaling [41]. The up-regulated expression of FoxE3 was achieved under the constitutive activation of Notch signaling by the forced expression of NICD, which may have induced a markedly larger increase in Notch signaling than that induced by *Rest* CKO in the present study. In any case, the expression of FoxE3 was only down-regulated by 2-fold by *Rest* CKO and this may be the reason that we did not detect impaired embryonic lens cell development. It is also important to note that RE1 sites have also been detected near the coding regions of the *E-cadherin*, *Crystallin A*, and *Crystallin B* genes [34], indicating that *Rest* is directly involved in lens fiber differentiation through the expression of these functional genes. Nevertheless, REST has a wide variety of target genes and may indirectly govern the necessary signaling pathway and each functional molecule in order to coordinate the proper cellular physiology required for functional lens fiber cells, and we observed changes in the expression of these candidates.

Since the cataract symptoms of *Sox1-Cre+*; *Rest*^2lox/2lox^ mice starts just after birth and obvious lens opacification is detected as early as one week after birth, the *Rest* CKO mouse is an uncommon cataract model. While cataracts have widely variable phenotypes [43], many of the genes responsible for congenital cataracts in humans are structural, such as the crystalline family and connexins or genes related to signal transduction. Although several transcription factors such as Pax6 [44], PITX3 [45], HSF4 [46], and HMX1 [47] were allelic with congenital cataracts, MAF family genes are well characterized for the indispensable transcription factor regulating lens development [48,49]. L-Maf/MafA is not required for normal mouse lens development [50], however, L-Maf/MafA and c-Maf are required for lens differentiation because they regulate crystallins [51,52]. Instead of increasing the number of lens epithelial cells by an aberrant cell specification process, the differentiation of lens fiber cells from precursor cells may be gradually impaired in *Rest* CKO lens and ultimately manifest as a cataract phenotype.

In conclusion, we herein demonstrated that *Rest* CKO mice showed a rare cataract phenotype, which severely affected lens differentiation. The deprivation of Rest from lens precursor cells caused a wide range of gene expression changes, including those for the Notch signaling pathway and lens differentiation and function, which eventually induced the unique cataract phenotype expressed from birth.

## Supporting Information

S1 Fig Immunohistochemical detection of lens marker proteins in *Rest*–deficient eyes *in vivo*. HE staining (A) and immunohistochemical staining with antibodies against Cryg (B), Prox1 (C), and c-Maf (D) at E15.5 (a and b), P0 (c and d), P7 (e and f), and 8W (g and h) in eyes from *Rest* CKO mice (b, d, f, h) and their control littermates (a, c, e, g). HE staining showed significant disorganization after birth (A). Cryg was strongly expressed in both genotypes at all time points (B). The expression levels of Prox1 and c-Maf in lenses were lower in *Rest* CKO mice than in the controls after birth (C and D). The scale bar represents 400 μm.
(TIF)

S2 Fig Expression of the control *β-actin* gene, genes related to Notch signaling, and lens-specification genes determined by quantitative RT-PCR. (A) *β-actin* expression for the quantitative control showed its constant expression in each developmental stage. (B-D) Expression of genes downstream of Notch signaling such as *Jagged1*, *Jagged2*, and *Dll1* in the *Rest*-deficient lens. (E-G) Expression of genes related to lens-specification *Pax6*, *Sox2 and Six3* in the *Rest*-deficient lens. Transcript levels were normalized to *β-actin*. Data are presented as average values with s.d. of more than three to fifteen independent samples. *; p<0.05, **; p<0.01.
(TIF)

S3 Fig Expression of cell cycle-related genes determined by quantitative RT-PCR. (A-F) Expression of cell cycle-related genes in various developmental stages. The expression of cyclin-dependent kinase-related genes, such as *p21*, *p27*, and *p57*, was significantly up-regulated in the lenses of *Rest* CKO mice, whereas that of *Cyclin D1* was not up-regulated. The expression of *c-Myc* and *K-Ras* was up-regulated in the *Rest* CKO lens. (G) *03B2-Catenin* expression was significantly up-regulated in the lenses of *Rest* CKO mice. Transcript levels were normalized to the level of *β-actin* in each sample. Data are presented as average values with s.d. of more than three to fifteen independent samples. *; p<0.05, **; p<0.01.
(TIF)

S4 Fig A gene expression analysis of the *Rest*-deficient lens and control lens having the *Sox1-Cre* transgene. The mRNA levels of *Rest* (A), *Rest4* (B), *Cre* (C), *Rest* target genes, *Tubb3* and *Syt4* (D and E), *Hes1* (F), *Sox1* (G) and *Sox2* (H) in *Rest*^+/+^, *Rest*^2lox/+^, and *Rest*^2lox/2lox^ allele with or without *Sox1-Cre* allele were measured by quantitative RT-PCR. +/+, 2lox/+, and 2lox/2lox indicate *Rest*^+/+^, *Rest*^2lox/+^, and *Rest*^2lox/2lox^ allele, respectively. Cre+ indicates the presence of single *Sox1-Cre* allele and Cre- indicates the absence of *Sox1-Cre* allele. Transcript levels were normalized to *β-actin*. Data are presented as average values with s.d. of more than three to fifteen independent samples. *; p<0.05, **; p<0.01.
(TIF)
