# Supplementary figures and images for: Disruption of Rest Leads to the Early Onset of Cataracts with the Aberrant Terminal Differentiation of Lens Fiber Cells
Source: PLoS One. 2016 Sep 15;11(9):e0163042. doi: 10.1371/journal.pone.0163042 (PMC5025245; doi:10.1371/journal.pone.0163042)

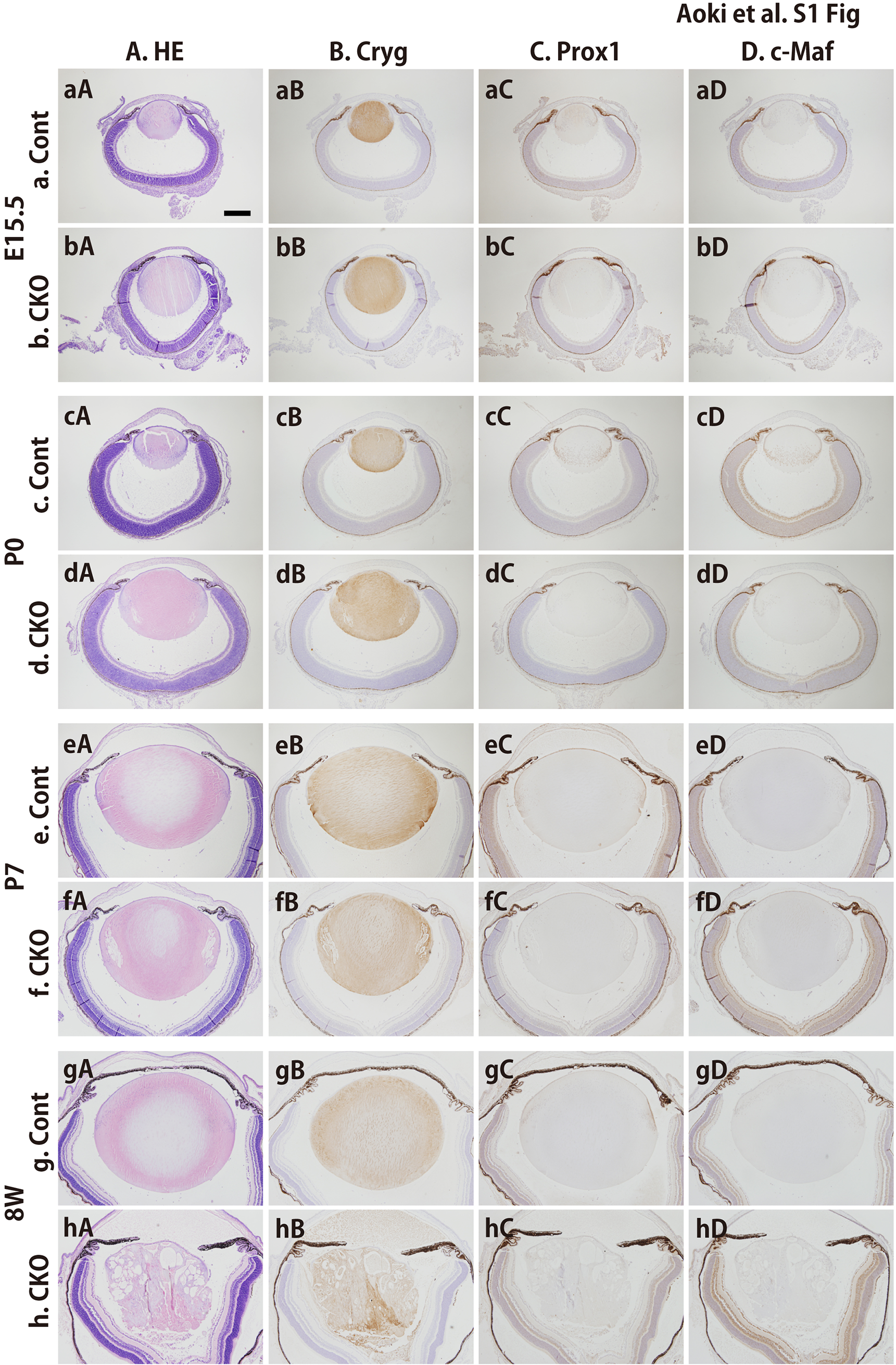

Supplement: S1 Fig — HE staining (A) and immunohistochemical staining with antibodies against Cryg (B), Prox1 (C), and c-Maf (D) at E15.5 (a and b), P0 (c and d), P7 (e and f), and 8W (g and h) in eyes from Rest CKO mice (b, d, f, h) and their control littermates (a, c, e, g). HE staining showed significant disorganization after birth (A). Cryg was strongly expressed in both genotypes at all time points (B). The expression levels of Prox1 and c-Maf in lenses were lower in Rest CKO mice than in the controls after birth (C and D). The scale bar represents 400 μm. (TIF) [file pone.0163042.s001.tif]

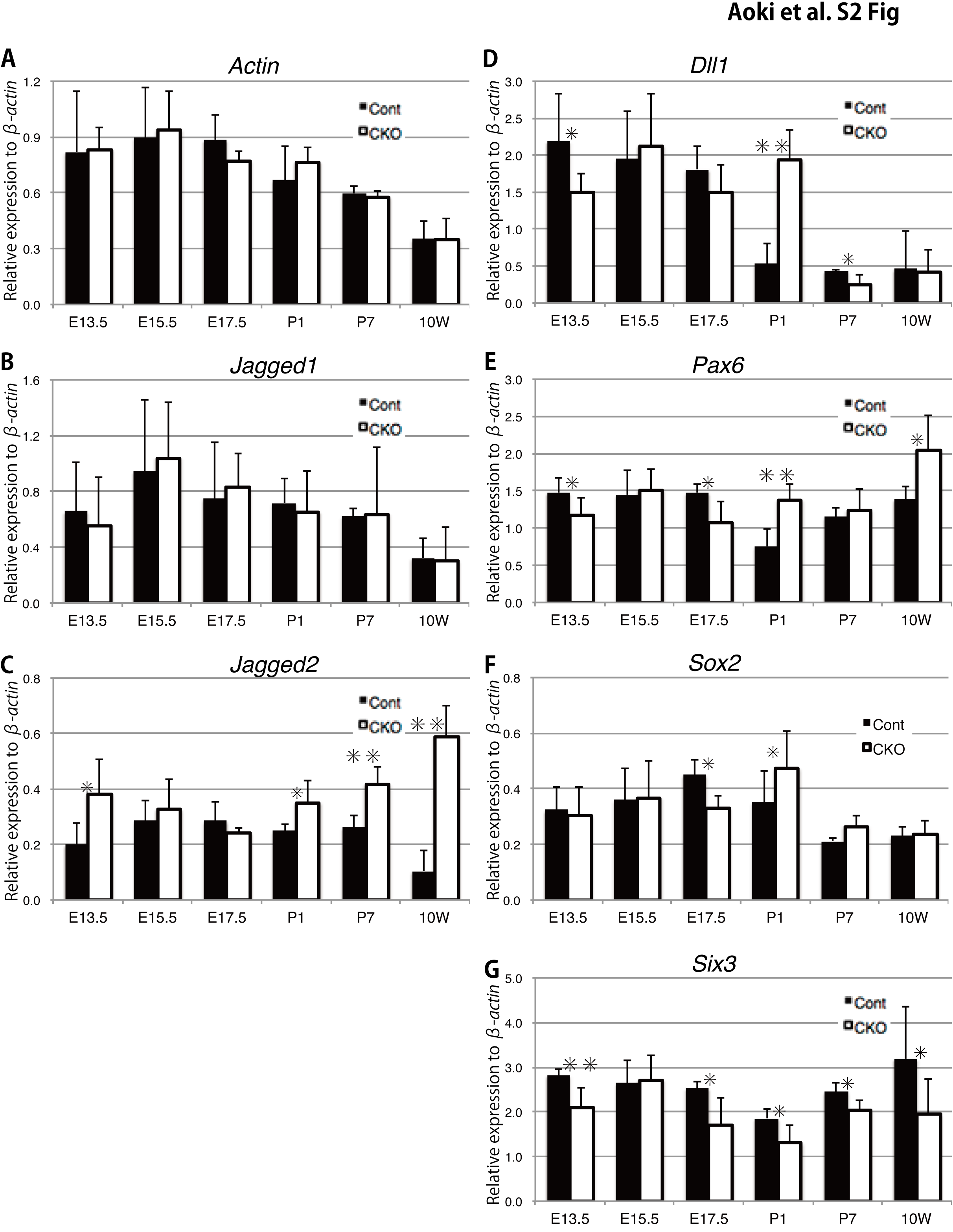

Supplement: S2 Fig — (A) β-actin expression for the quantitative control showed its constant expression in each developmental stage. (B-D) Expression of genes downstream of Notch signaling such as Jagged1, Jagged2, and Dll1 in the Rest-deficient lens. (E-G) Expression of genes related to lens-specification Pax6, Sox2 and Six3 in the Rest-deficient lens. Transcript levels were normalized to β-actin. Data are presented as average values with s.d. of more than three to fifteen independent samples. *; p<0.05, **; p<0.01. (TIF) [file pone.0163042.s002.tif]

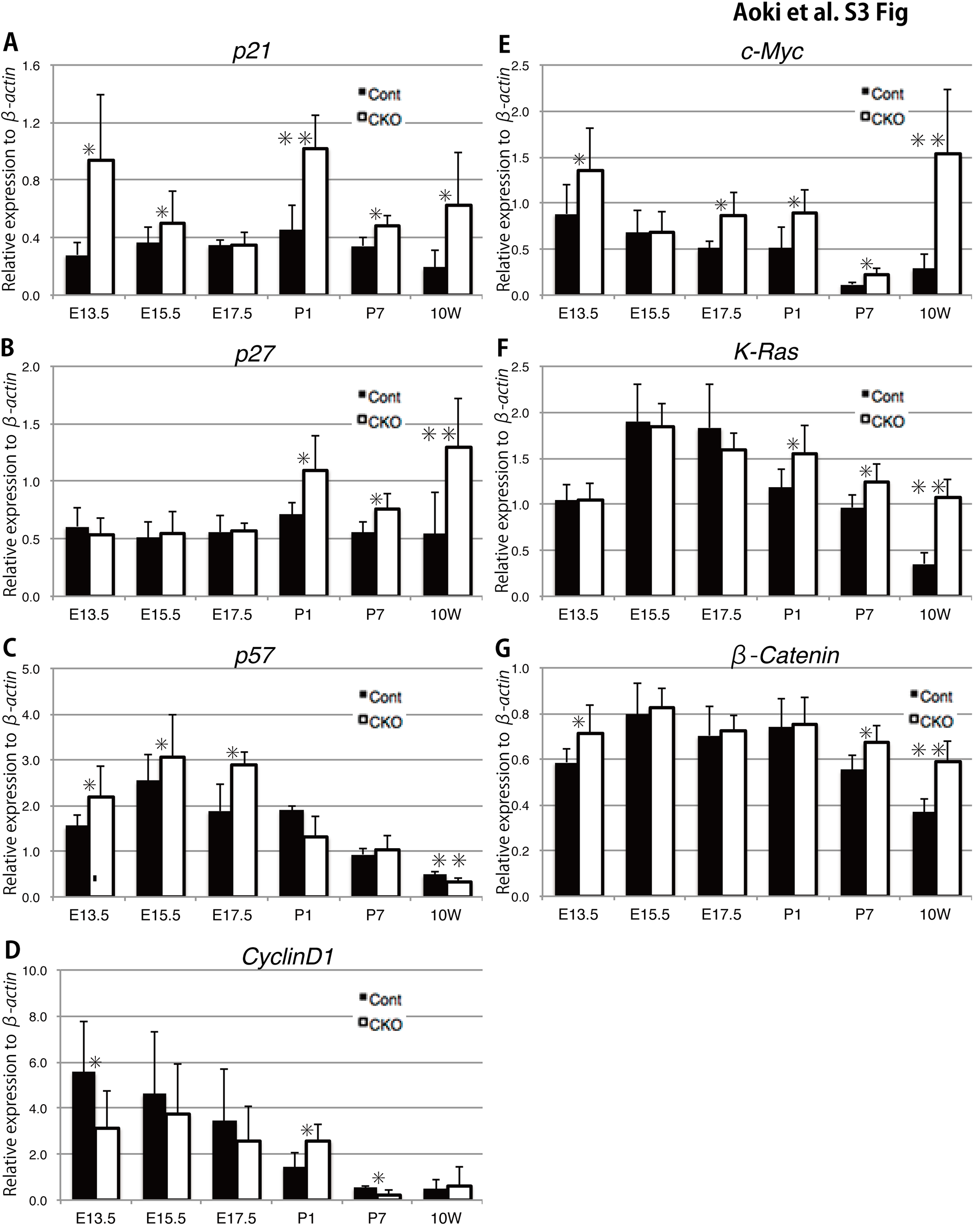

Supplement: S3 Fig — (A-F) Expression of cell cycle-related genes in various developmental stages. The expression of cyclin-dependent kinase-related genes, such as p21, p27, and p57, was significantly up-regulated in the lenses of Rest CKO mice, whereas that of Cyclin D1 was not up-regulated. The expression of c-Myc and K-Ras was up-regulated in the Rest CKO lens. (G) 03B2-Catenin expression was significantly up-regulated in the lenses of Rest CKO mice. Transcript levels were normalized to the level of β-actin in each sample. Data are presented as average values with s.d. of more than three to fifteen independent samples. *; p<0.05, **; p<0.01. (TIF) [file pone.0163042.s003.tif]

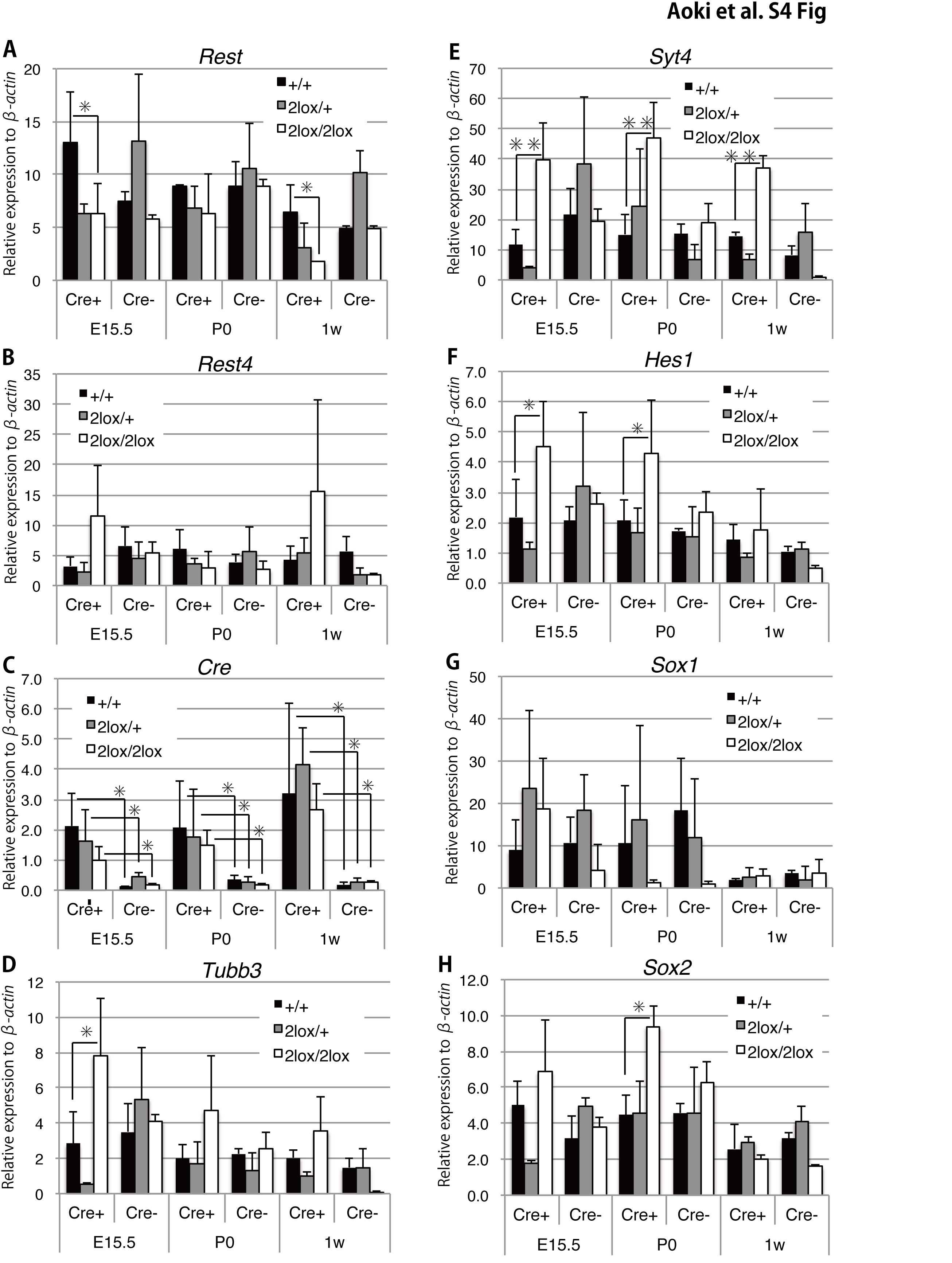

Supplement: S4 Fig — The mRNA levels of Rest (A), Rest4 (B), Cre (C), Rest target genes, Tubb3 and Syt4 (D and E), Hes1 (F), Sox1 (G) and Sox2 (H) in Rest+/+, Rest2lox/+, and Rest2lox/2lox allele with or without Sox1-Cre allele were measured by quantitative RT-PCR. +/+, 2lox/+, and 2lox/2lox indicate Rest+/+, Rest2lox/+, and Rest2lox/2lox allele, respectively. Cre+ indicates the presence of single Sox1-Cre allele and Cre- indicates the absence of Sox1-Cre allele. Transcript levels were normalized to β-actin. Data are presented as average values with s.d. of more than three to fifteen independent samples. *; p<0.05, **; p<0.01. (TIF) [file pone.0163042.s004.tif]
